# Behaviour change intervention for inhaler disposal, Spain 

**DOI:** 10.2471/BLT.25.294976

**Published:** 2026-04-16

**Authors:** Noé Garin, Borja Zarate-Tamames, Esther Vaquero-Alvarez, Paula Gomez-Rivas, Sonia Jornet-Montaña, Jorge Del Estal-Jimenez, Alvaro Narrillos-Moraza, M Angeles Lopez-Montenegro, Saioa Domingo-Echaburu

**Affiliations:** a Pharmacy Department, Hospital de la Santa Creu i Sant Pau, Sant Antoni Maria Claret 167, 08025 Barcelona, Spain.; b Pharmacy Department, Hospital San Juan de Dios de Córdoba, Cordoba, Spain.; c Pharmacy Department, Hospital Universitario Miguel Servet, Zaragoza, Spain.; d Pharmacy Department, Hospital Universitari de Tarragona Joan XXIII, Tarragona, Spain.; e Pharmacy Department, Parc Taulí Hospital Universitari, Universitat Autònoma de Barcelona, Sabadell, Spain.; f Pharmacy Department, Hospital General Universitario Gregorio Marañón, Madrid, Spain.; g Pharmacy Department, Hospital Lluís Alcanyís de Xàtiva, Valencia, Spain.; h Pharmacy Service of Debagoiena Integrated Health Organisation, Osakidetza Basque Health Service, Gipuzkoa, Spain.

## Abstract

**Objective:**

To analyse metered-dose inhaler disposal behaviour in asthma patients, determine the patient characteristics that contribute to appropriate disposal and quantify the effect of a pharmacy-led educational intervention.

**Methods:**

We conducted a multicentre cross-sectional study in 40 Spanish hospitals followed by a pre–post quasi-experimental evaluation. We surveyed adults with uncontrolled severe asthma receiving biological therapy to determine sociodemographic, health, environmental attitude and disposal behaviour characteristics. At the time of biological therapy dispensation, we provided patients with an infographic on inhaler environmental impact and appropriate disposal. We resurveyed participants 3 months later to assess changes in knowledge, attitude and disposal behaviour. We identified factors associated with correct inhaler disposal and quantified the impact of the intervention using a multivariate logistic regression and McNemar test, respectively.

**Findings:**

Despite 86.7% (435/502) of our study participants valuing environmental conservation and 91.6% (460/502) being aware of medication disposal programmes, only 46.6% (234/502) correctly disposed of inhalers. Factors associated with appropriate disposal included practising household recycling, high perception of inhaler environmental impact, awareness of medication disposal programme, low income and self-reported limitations to daily activities. On resurveying patients 3 months after our pharmacy-led intervention, we observed an increase in correct inhaler disposal to 84.1% (418/497) with parallel gains in awareness and recycling behaviours.

**Conclusion:**

We revealed a discrepancy between the environmental concerns of asthma patients and inhaler disposal behaviour, due to limited knowledge of inhaler environmental impact. We demonstrated that this knowledge gap can be reduced, doubling the correct disposal of inhalers, through a targeted pharmacy-led intervention.

## Résumé

**Objectif:**

Analyser les comportements en matière d’élimination des aérosols-doseurs chez les patients asthmatiques, déterminer les caractéristiques des patients qui favorisent une élimination appropriée et quantifier l’effet d’une intervention éducative organisée en pharmacie.

**Méthodes:**

Nous avons mené une étude transversale multicentrique (dans 40 hôpitaux espagnols) suivie d’une pré-évaluation et d’une post-évaluation quasiexpérimentales. Nous avons interrogé des adultes atteints d’asthme grave non contrôlé qui reçoivent un traitement biologique afin de déterminer leurs caractéristiques sur le plan sociodémographique et en matière de santé, d’attitudes environnementales et de comportements concernant l’élimination des aérosols-doseurs. Au moment de la délivrance du traitement biologique, nous avons remis aux patients une infographie sur l’impact environnemental des aérosols et leur élimination appropriée. Nous avons interrogé à nouveau les participants trois mois plus tard afin d’évaluer les changements en matière de connaissances, d’attitudes et de comportements d’élimination. Nous avons identifié les facteurs associés à l’élimination correcte des aérosols et quantifié l’impact de l’intervention à l’aide d’une régression logistique multivariée et du test de McNemar, respectivement.

**Résultats:**

Bien que 86,7% (435/502) des participants à notre étude accordent de l’importance à la protection de l’environnement et que 91,6% (460/502) connaissent l’existence de programmes de collecte des médicaments usagés, seuls 46,6% (234/502) ont éliminé correctement leurs aérosols-doseurs. Les facteurs associés à une élimination appropriée comprenaient la pratique du recyclage domestique, une perception élevée de l’impact environnemental des aérosols-doseurs, la connaissance des programmes d’élimination des médicaments, un faible revenu et des limitations autodéclarées dans les activités quotidiennes. Lors d’une nouvelle enquête menée auprès des patients trois mois après notre intervention organisée en pharmacie, nous avons observé une augmentation à 84,1% (418/497) du taux d’élimination correcte des aérosols-doseurs, accompagnée d’une amélioration parallèle de la sensibilisation et des comportements de recyclage.

**Conclusion:**

Nous avons mis en évidence un décalage entre les préoccupations environnementales des patients asthmatiques et leur comportement en matière d’élimination des aérosols-doseurs. Cet écart est dû à une connaissance limitée de l’impact environnemental de ces dispositifs. Nous avons démontré qu’il était possible de combler ce déficit de connaissances, doublant ainsi le taux d’élimination correcte des aérosols-doseurs, grâce à une intervention ciblée organisée en pharmacie.

## Resumen

**Objetivo:**

Analizar el comportamiento de eliminación de inhaladores de dosis medida en pacientes con asma, determinar las características de los pacientes que contribuyen a una eliminación adecuada y cuantificar el efecto de una intervención educativa liderada por farmacia.

**Métodos:**

Se realizó un estudio transversal multicéntrico (40 hospitales españoles), seguido de una evaluación cuasiexperimental antes y después de la intervención. Se encuestó a personas adultas con asma grave no controlada que recibían terapia biológica para determinar características sociodemográficas, de salud, de actitud ambiental y de comportamiento de eliminación. En el momento de la dispensación de la terapia biológica, se proporcionó a los pacientes una infografía sobre el impacto ambiental de los inhaladores y su eliminación adecuada. Se volvió a encuestar a los participantes tres meses después para evaluar los cambios en el conocimiento, la actitud y el comportamiento de eliminación. Se identificaron los factores asociados a la eliminación correcta de los inhaladores y se cuantificó el impacto de la intervención mediante regresión logística multivariante y la prueba de McNemar, respectivamente.

**Resultados:**

A pesar de que el 86,7% (435/502) de los participantes valoraba la conservación del medio ambiente y el 91,6% (460/502) conocía los programas de eliminación de medicamentos, solo el 46,6% (234/502) eliminaba correctamente los inhaladores. Los factores asociados a una eliminación adecuada incluyeron la práctica de reciclaje doméstico, una alta percepción del impacto ambiental de los inhaladores, el conocimiento de los programas de eliminación de medicamentos, bajos ingresos y limitaciones en las actividades diarias notificadas por los propios pacientes. Al volver a encuestar a los pacientes tres meses después de la intervención liderada por farmacia, se observó un aumento de la eliminación correcta de inhaladores hasta el 84,1% (418/497), junto con mejoras paralelas en el conocimiento y en los comportamientos de reciclaje.

**Conclusión:**

Se evidenció una discrepancia entre la preocupación ambiental de los pacientes con asma y el comportamiento de eliminación de los inhaladores, debida a un conocimiento limitado de su impacto ambiental. Se demostró que esta brecha de conocimiento puede reducirse, duplicando la eliminación correcta de los inhaladores, mediante una intervención específica liderada por farmacia.

## ملخص

**الغرض:**

تحليل سلوك التخلص من أجهزة الاستنشاق ذات الجرعات المقننة لدى مرضى الربو، وتحديد خصائص المرضى التي تُسهم في التخلص السليم منها، وقياس أثر برنامج تعليمي تحت إشراف الصيدليات.

**الطريقة:**

أجرينا دراسة متعددة المراكز (40 مستشفى في أسبانيا) شملت عدة قطاعات، تلتها دراسة شبه تجريبية قبلية وبعدية. قمنا بمسح آراء البالغين المصابين بنوبات الربو الحاد غير الخاضعة للسيطرة، والذين يتلقون العلاج البيولوجي لتحديد خصائصهم الاجتماعية والسكانية والصحية والبيئية، بالإضافة إلى سلوكياتهم المتعلقة بالتخلص من أجهزة الاستنشاق. عند صرف العلاج البيولوجي، قمنا بتزويد المرضى برسم توضيحي حول الأثر البيئي لأجهزة الاستنشاق والتخلص السليم منها. قمنا بإجراء مسح آخر للمشاركين بعد 3 أشهر لتقييم التغيرات في المعرفة والمواقف وسلوكيات التخلص من أجهزة الاستنشاق. قمنا بتحديد العوامل المرتبطة بالتخلص الصحيح من أجهزة الاستنشاق، وقمنا بقياس أثر التدخل باستخدام التحوف اللوجستي متعدد المتغيرات واختبار McNemar، على الترتيب.

**النتائج:**

على الرغم من أن %86.7 (435/502) من المشاركين في دراستنا يُقدّرون الحفاظ على البيئة، وأن %91.6 (460/502) على دراية ببرامج التخلص من الأدوية، إلا أن %46.6 فقط (234/502) تخلصوا من أجهزة الاستنشاق بشكل صحيح. وشملت العوامل المرتبطة بالتخلص السليم ممارسة إعادة التدوير المنزلي، والإدراك المتقدم للأثر البيئي لأجهزة الاستنشاق، والوعي ببرنامج التخلص من الأدوية، وانخفاض الدخل، والقيود التي أبلغ عنها المشاركون أنفسهم في أنشطتهم اليومية. وعند إجراء مسح آخر لآراء المرضى بعد 3 أشهر من تدخلنا تحت إشراف الصيدليات، لاحظنا زيادة في التخلص الصحيح من أجهزة الاستنشاق إلى %84.1 (418/497)، مع ارتفاعات موازية في الوعي وسلوكيات إعادة التدوير.

**الاستنتاج:**

قمنا بالكشف عن الفارق بين المخاوف البيئية لدى مرضى الربو وسلوكياتهم في التخلص من أجهزة الاستنشاق، وذلك بسبب معرفتهم المحدودة بالأثر البيئي لهذه الأجهزة. لقد أوضحنا أنه يمكن تقليص هذه الفجوة المعرفية، ومضاعفة التخلص الصحيح من أجهزة الاستنشاق، وذلك من خلال تدخل مُوجّه تحت إشراف الصيدليات.

## 摘要

**目的:**

旨在分析哮喘患者定量雾化吸入器的处置行为，明确影响规范处置行为的患者特征，并量化由药房主导的教育干预措施所产生的效果。

**方法:**

我们开展了多中心（40 家西班牙医院）横断面研究，并随后开展前后对照试验评估。我们选取接受生物制剂疗法的重度哮喘控制不佳的成年患者开展调研，分析其社会人口学、健康状况、对环境的态度以及废弃处置行为等特征。在配发生物治疗药物时，我们向患者发放科普图解，介绍吸入器对环境的影响以及规范的处置方式。我们在 3 个月后对参与者进行再次调研，评估其在认知、态度和处置行为方面的变化。我们分别采用多因素逻辑回归和麦克尼马尔检验，筛选出与正确处置吸入器行为有关的影响因素，并对干预措施的实施效果进行量化。

**结果:**

尽管研究中，86.7% (435/502) 的参与者重视环境保护，且 91.6% (460/502) 知晓药品处置项目，但只有 46.6% (234/502) 能够正确处置吸入器。与规范处置行为有关的因素包括：家庭垃圾分类回收习惯、充分认识到吸入器对环境的危害、知晓药品回收处置项目、低收入以及自述受到日常活动的限制。在开展药房主导的干预措施并于 3 个月后对患者进行回访调研之后，我们发现，正确处置吸入器的概率提升至 84.1% (418/497)，患者的认知水平和回收处置行为也同步有所改善。

**结论:**

我们发现，哮喘患者的环保意识与其吸入器处置行为之间存在偏差，主要原因是患者对吸入器环境危害的认知不足。研究结果表明，通过药房主导的针对性干预措施，可有效缩小患者的认知差距，使吸入器的规范处置率实现翻倍提升。

## Резюме

**Цель:**

Проанализировать подходы пациентов с астмой к утилизации дозированных аэрозольных ингаляторов, определить характеристики пациентов, предрасполагающие к правильной утилизации, а также оценить эффективность обучающих инициатив аптек.

**Методы:**

Авторы провели многоцентровое (в 40 испанских больницах) поперечное исследование с последующей квазиэкспериментальной оценкой по схеме «до и после». Были опрошены взрослые пациенты с тяжелой неконтролируемой астмой, получающие биологическую терапию. Цель такого опроса заключалась в установлении их социально-демографического статуса, состояния здоровья, отношения к окружающей среде и способов утилизации ингаляторов. Во время отпуска препаратов биологической терапии пациентам предоставили инфографику о воздействии ингаляторов на окружающую среду и правильной утилизации. Через 3 месяца участников повторно опросили, чтобы оценить изменения в уровне знаний, отношении к проблеме и подходе к утилизации. Авторы выявили факторы, связанные с правильной утилизацией ингаляторов, и количественно оценили влияние вмешательства с использованием многомерной логистической регрессии и критерия Мак-Немара соответственно.

**Результаты:**

Несмотря на то что 86,7% (435 из 502) участников исследования придавали значение охране окружающей среды, а 91,6% (460 из 502) знали о программах утилизации лекарственных средств, только 46,6% (234 из 502) правильно утилизировали ингаляторы. Факторы, связанные с правильной утилизацией: практика раздельного сбора бытовых отходов, высокая степень осознания воздействия ингаляторов на окружающую среду, осведомленность о программе утилизации лекарственных средств, низкий доход и самостоятельно сообщаемые ограничения в повседневной активности. При повторном опросе пациентов через 3 месяца после проведения обучающих инициатив аптек было отмечено увеличение доли правильной утилизации ингаляторов до 84,1% (418 из 497 случаев) наряду с ростом осведомленности и улучшением подхода к переработке отходов.

**Вывод:**

Авторы выявили несоответствие между экологической обеспокоенностью пациентов с астмой и их подходом к утилизации ингаляторов, что обусловлено ограниченными знаниями о воздействии ингаляторов на окружающую среду. Было продемонстрировано, что этот пробел в знаниях можно сократить, удвоив уровень правильной утилизации ингаляторов, с помощью целенаправленных обучающих инициатив аптек.

## Introduction

The surface temperature of our planet has increased over the last decades, with both 2023 and 2024 registering the highest average values recorded.^1^ Specifically, the global average temperature in 2024 was 1.55 °C above preindustrial levels (1850–1900), surpassing the critical threshold of 1.5 °C, a level linked to extreme weather events including floods, droughts and heatwaves.^2^ Environmental factors, such as air pollution, poor water quality and climate change, are a significant threat to global health, accounting for an estimated 24% of mortality worldwide.^3^ According to estimates from the World Health Organization (WHO), climate change is projected to contribute an additional 250 000 deaths annually between 2030 and 2050. This increased mortality will be the result of the coexistence of climate-related hazards, vulnerability factors and exposure pathways, leading to an increase in respiratory diseases, waterborne illnesses, zoonoses, malnutrition, injuries, noncommunicable diseases and mental health conditions.^3^

The health-care sector contributes significantly to global carbon emissions, accounting for an estimated 5.5% of the global carbon footprint, with some countries, such as Australia and United States of America (USA), reaching up to 7–10%.^4^ If health care was considered a country, it would rank fifth after China, USA, India and Russian Federation in terms of carbon emissions.^5^ The manufacture of pharmaceuticals alone represents 25–50% of the health-care-related carbon footprint of Europe, according to data from France, the Kingdom of the Netherlands and the United Kingdom of Great Britain and Northern Ireland.^6^^–^^8^ Among the prescribed medicines used globally, pressurized metered-dose inhalers make a significant contribution to the emission of greenhouse gases because of their use of hydrofluoroalkane propellants (HFA). In particular, HFA-134a and HFA-227ea have a global warming potential (defined as the impact a gas will have on atmospheric warming, relative to carbon dioxide) of 1300 and 3350, respectively.^9^

Spain has one of the highest proportions of pressure metered-dose inhalers in the WHO European Region, comprising 52% of all inhaler prescriptions.^10^ This high proportion results in a self-perpetuating cycle: for every degree Celsius above an average temperature of 29 °C, there is an approximate 7% increase in mortality and a 4% rise in hospital admissions because of respiratory problems.^11^^,^^12^ Although achieving a more sustainable selection of inhalers is a key priority, a group of patients will inevitably continue to require pressurized metered-dose inhalers because of their specific clinical characteristics. 

In addition to the negative impact in terms of greenhouse gas emissions, other detrimental effects associated with all types of inhalers include the high energy consumption required for metal production, the environmental consequences of plastic waste from inhaler devices, and deforestation linked to the cardboard and paper used in packaging and leaflets. Indeed, several studies have demonstrated the high environmental impact of inhalers when considering variables such as material depletion, acidification, water eutrophication, ecotoxicity, health impact, ozone layer depletion and photochemical oxidant formation.^13^ Fostering initiatives that promote the proper management of inhaler waste is therefore imperative, where patient education and cooperation can play a pivotal role. 

In Spain, a nationwide disposal programme for household pharmaceutical waste (*Sistema Integrado de Gestión y Recogida de Envases*, known as SIGRE) is accessible at community pharmacies. However, the available evidence suggests notable gaps in patient awareness and inhaler recycling practice.^14^

The aim of our study was to conduct an analysis of inhaler disposal patterns in adult patients with uncontrolled severe asthma, specifically to (i) evaluate the prevalence of appropriate inhaler disposal at community pharmacies; (ii) determine whether certain sociodemographic or health characteristics contribute to particular inhaler recycling patterns; and (iii) investigate the effectiveness of an informational intervention by pharmacists, including by an educational infographic, on the correct disposal of inhaler waste.

## Methods

### Design

We conducted an analytical cross-sectional study followed by a single-arm pre–post quasi-experimental evaluation on an educational intervention to investigate the management of inhaler waste in uncontrolled severe asthma patients receiving biological treatment in clinical settings. We conducted our study across 40 hospitals in Spain (online repository),^15^ encompassing institutions of varying complexity to ensure broad geographical representation, generalizability and applicability of our findings.

### Study population

We enrolled patients to our study during March–November 2024 as their asthma biological therapy was being dispensed, either in the outpatient pharmacy or day-hospital settings (online repository).^15^ We considered adults aged 18 years or older with a diagnosis of uncontrolled severe asthma, who were receiving biological treatment for asthma, and able to read, comprehend and complete the study questionnaires, as eligible. 

### Procedure

We asked patients to complete a brief survey, including questions on sociodemographic, such as marital, status, level of education, smoking status, type of housing, income and occupational status, and health characteristics. We also asked about their asthma management, such as asthma control test score, number of exacerbations in the past 12 months and current use of systemic corticosteroids. We collected additional clinical information from electronic health records. We also asked participants about their perception of environmental issues, adherence to inhaler usage and their current practices for inhaler waste disposal (online repository).^15^ Our primary variable of interest was the method of inhaler disposal, with the options being with regular waste (that is, inappropriate disposal) or via the pharmacy disposal programme. 

**Fig. 1:**
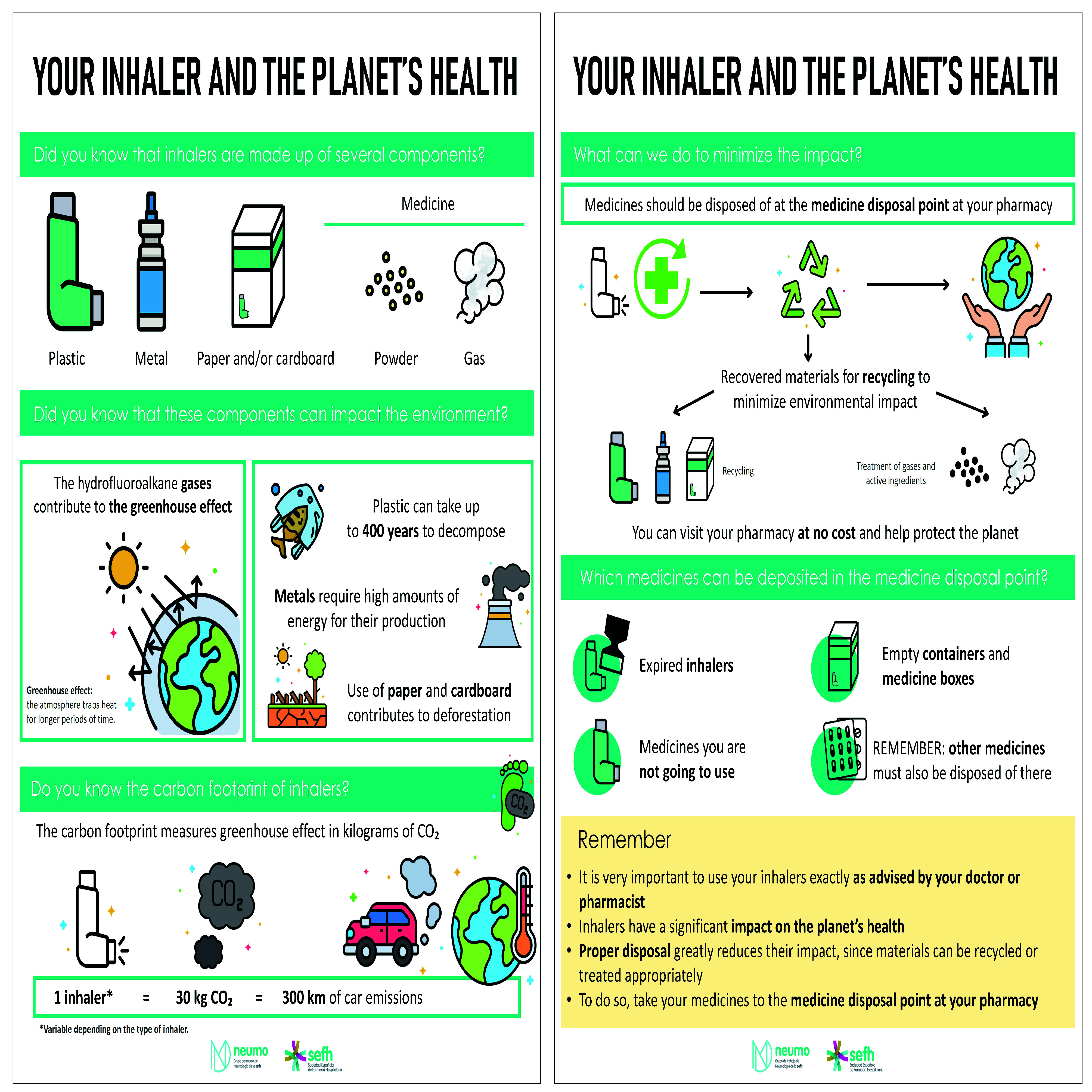
Educational infographic illustrating the environmental impact of inhalers, with guidelines for their proper disposal at community pharmacies, Spain, March–November 2024

Note: the infographic in Spanish is available in the online repository.^15^

We provided each participant with an educational infographic outlining the environmental impact of inhalers and proper waste management procedures, which was further explained by the pharmacy team (Fig. 1; online repository).^15^

We recontacted participants 3 months later by telephone to complete a follow-up survey, assessing any changes in knowledge, attitudes and behaviour regarding inhaler disposal. We recorded all survey responses, a minimal set of baseline clinical data, such as asthma history and treatment details, and follow-up survey responses using the Research Electronic Data Capture software (Vanderbilt University, Nashville, USA).

### Sample size

Based on previous research^14^ that reported the correct disposal of inhalers by 42.9% (130/303) of study participants, we assumed a starting prevalence of correct inhaler disposal of 40% for the purposes of calculating our required sample size. Using a 5% precision and a 95% confidence interval (CI), we calculated a minimum sample size of 369 participants. To assess the impact of the educational intervention, we aimed to evaluate changes in inhaler disposal practices. Assuming a baseline prevalence of 40% and an expected improvement to 50%, we recalculated the minimum sample size as 394 participants to ensure sufficient power for both primary (prevalence) and secondary (improvement after intervention) objectives.

### Statistical analysis

We used descriptive statistics to summarize baseline characteristics and survey responses, including frequencies, proportions, means and standard deviations. We reported the prevalence of correct inhaler disposal as both a count and percentage, with 95% CI. We used the *χ^2^*, Fisher exact and student *t* tests to measure differences in the prevalence of sociodemographic, health and environmental variables between the groups disposing of inhalers correctly versus incorrectly.

We conducted univariate and multivariate logistic regression analyses to explore the relationship between (i) sociodemographic, clinical and environmental variables; and (ii) correct disposal practices. The multivariate logistic regression model included those variables with an association in the bivariate analyses defined as *P*-value: < 0.1. We fitted additional regression models for combinations of variables to test whether potential theoretical interactions were present with regards to the dependent variable, that is, correct disposal of inhalers. We also tested multicollinearity using the condition index, a measure of dependency among variables (with values > 20 indicating potential collinearity), and the variance inflation factor (with values > 10 suggesting problematic correlation between variables). 

We evaluated the impact of the educational intervention using paired comparisons (before versus after) of correct disposal rates using the McNemar test. We performed all data analysis using SPSS software, version 20.0 (IBM, New York, USA).

### Ethics

Our study was approved by the reference Clinical Research Ethics Committee: Hospital de la Santa Creu i Sant Pau, Barcelona, Spain, reference no. EC/23/195/7301 (R-OBS), 17 May 2023. All investigators conducted themselves according to the local legislation, institutional requirements and the principles expressed in the Declaration of Helsinki. All participants provided written informed consent.

## Results

### Descriptive statistics

We enrolled a total of 502 patients (94.9% of 529 patients originally approached); 497 completed the 3-month follow-up (99.0% retention). Our study participants had a mean age of 56.4 years (standard deviation, SD: 14.5), 332 (66.1%) of whom were women and 466 (92.8%) were non-smokers. Most participants reported poor general health (312; 62.2%) and moderate-to-severe limitations in daily activities (370; 73.7%). Regarding comorbidities, 331 (65.9%) reported respiratory comorbidities, and 360 (71.7%) reported other comorbidities. A total of 345 (68.7%) of the participants reported maintenance use of pressurized metered-dose inhalers, 331 (65.9%) dry powder inhalers and 133 (26.5%) soft mist inhalers. On average, patients reported having 2.1 (SD: 0.6) inhalers in use. Medication adherence was high, with 472 (94.0%) of patients reporting good adherence. Regarding asthma control, 259 (51.6%) achieved good control (asthma control test score ≥ 20), although 194 (38.6%) perceived their control as moderate or low. Additionally, 263 (52.4%) participants reported experiencing at least one exacerbation in the past year. The full list of sociodemographic and clinical characteristics is available in Table 1.

**Table 1:** Participant characteristics and multivariate logistic regression results, in study of appropriate inhaler disposal, Spain, March–November 2024

Characteristic	No. (%) (*n* = 502)^a^				Correlates of appropriate inhaler disposal by multivariate logistic regression	
	Inappropriate disposal of inhalers	Recycling of inhalers through disposal programme^b^	Total		Crude OR (95% CI)	aOR (95% CI)^c^
**Sociodemographic **						
Sex						
Male	103 (20.5)	67 (13.3)	170 (33.9)		Reference	Reference
Female	165 (32.9)	167 (33.3)	332 (66.1)		1.556 (1.069–2.265)	1.089 (0.718–1.651)
Mean age (SD), years	55.3 (14.3)	57.5 (14.7)	56.4 (14.5)		1.011 (0.998–1.023)	1.008 (0.992–1.024)
Smoking status						
Non-smoker	250 (49.8)	216 (43.0)	466 (92.8)		Reference	ND
Smoker	18 (3.6)	18 (3.6)	36 (7.2)		1.157 (0.587–2.281)	ND
Marital status						
Single	90 (17.9)	76 (15.1)	166 (33.1)		Reference	ND
Married or cohabiting	178 (35.5)	158 (31.5)	336 (66.9)		1.051 (0.724–1.526)	ND
Level of education						
Primary or lower	76 (15.1)	74 (14.7)	150 (29.9)		Reference	ND
Secondary	94 (18.7)	69 (13.7)	163 (32.5)		0.754 (0.483–1.178)	ND
University or higher	98 (19.5)	91 (18.1)	189 (37.6)		0.954 (0.621–1.464)	ND
Employment status						
Unemployed	137 (27.3)	144 (28.7)	281 (56.0)		Reference	Reference
Employed	131 (26.1)	90 (17.9)	221 (44.0)		0.654 (0.458–0.933)	0.791 (0.992–1.024)
Type of housing						
Rented or other	45 (9.0)	53 (10.6)	98 (19.5)		Reference	ND
Owned or family-owned	223 (44.4)	181 (36.1)	404 (80.5)		0.689 (0.442–1.073)	ND
Household income						
Low	20 (4.0)	33 (6.6)	53 (10.6)		2.036 (1.133–3.657)	2.628 (1.317–5.241)
Sufficient	248 (49.4)	201 (40.0)	449 (89.4)		Reference	Reference
**Health**						
General health						
Poor	166 (33.1)	146 (29.1)	312 (62.2)		Reference	ND
Good	102 (20.3)	88 (17.5)	190 (37.8)		0.981 (0.683–1.408)	ND
Health problems limiting daily activities						
None	83 (16.5)	49 (9.8)	132 (26.3)		Reference	Reference
Moderate to severe	185 (36.9)	185 (36.9)	370 (73.7)		1.694 (1.127–2.546)	1.639 (1.057–2.540)
Respiratory comorbidities						
No	87 (17.3)	84 (16.7)	171 (34.1)		Reference	ND
Yes	181 (36.1)	150 (29.9)	331 (65.9)		0.858 (0.593–1.242)	ND
Other comorbidities						
No	81 (16.1)	61 (12.2)	142 (28.3)		Reference	ND
Yes	187 (37.3)	173 (34.5)	360 (71.7)		1.228 (0.831–1.817)	ND
Number of inhalers in use (SD)	2.13 (0.62)	2.08 (0.69)	2.10 (0.60)		0.900 (0.689–1.175)	ND
Dry powder inhaler use						
No	85 (16.9)	86 (17.1)	171 (34.1)		NA	NA
Yes	183 (36.5)	148 (29.5)	331 (65.9)		NA	NA
Pressurized metered-dose inhaler use						
No	90 (17.9)	67 (13.3)	157 (31.3)		NA	NA
Yes	178 (35.5)	167 (33.3)	345 (68.7)		NA	NA
Soft mist inhaler use						
No	207 (41.2)	162 (32.3)	369 (73.5)		NA	NA
Yes	61 (12.2)	72 (14.3)	133 (26.5)		NA	NA
Chronic oral glucocorticosteroids						
No	235 (46.8)	197 (39.2)	432 (86.1)		Reference	ND
Yes	33 (6.6)	37 (7.4)	70 (13.9)		1.337 (0.806–2.219)	ND
Other medication						
No	92 (18.3)	71 (14.1)	163 (32.5)		Reference	ND
Yes	176 (35.1)	163 (32.5)	339 (67.5)		1.200 (0.824–1.748)	ND
Adherence to medication						
Good	253 (50.4)	219 (43.6)	472 (94.0)		Reference	ND
Poor	15 (3.0)	15 (3.0)	30 (6.0)		1.155 (0.552–2.417)	ND
Asthma control test score						
Good (≥ 20)	137 (27.3)	122 (24.3)	259 (51.6)		Reference	ND
Poor (< 20)	131 (26.1)	112 (22.3)	243 (48.4)		0.960 (0.676–1.364)	ND
Asthma control perception						
Moderate to low	101 (20.1)	93 (18.5)	194 (38.6)		Reference	ND
Good to very good	167 (33.3)	141 (28.1)	308 (61.4)		0.917 (0.640–1.314)	ND
Exacerbations within past 12 months						
No	128 (25.5)	111 (22.1)	239 (47.6)		Reference	ND
Yes	140 (27.9)	123 (24.5)	263 (52.4)		1.013 (0.713–1.439)	ND
**Environmental**						
Importance given to environmental conservation						
Low	40 (8.0)	27 (5.4)	67 (13.3)		0.743 (0.441–1.255)	ND
High	228 (45.4)	207 (41.2)	435 (86.7)		Reference	ND
Belief that pollution and climate change affect health						
No	89 (17.7)	62 (12.4)	151 (30.1)		Reference	ND
Yes	179 (35.7)	172 (34.3)	351 (69.9)		1.379 (0.038–2.029)	ND
Practising separation of general waste for recycling						
No	63 (12.5)	23 (4.6)	86 (17.1)		Reference	Reference
Yes	205 (40.8)	211 (42.0)	416 (82.9)		2.819 (1.685–4.717)	2.823 (1.617–4.926)
Perception of environmental impact of inhaler						
Low	164 (32.7)	113 (22.5)	277 (55.2)		Reference	Reference
High	104 (20.7)	121 (24.1)	225 (44.8)		1.689 (1.184–2.408)	1.637 (1.104–2.428)
Perception of environmental impact of inhalers relative to other medications						
Lower	187 (37.3)	149 (29.7)	336 (66.9)		Reference	ND
Higher	80 (15.9)	85 (16.9)	165 (32.9)		1.333 (0.918–1.937)	ND
Missing	1 (0.2%)	NA	1 (0.2)		ND	ND
Awareness of medication disposal programme^b^						
No	41 (8.2)	1 (0.2)	42 (8.4)		Reference	Reference
Yes	227 (45.2)	233 (46.4)	460 (91.6)		42.084 (5.740–308.520)	37.786 (5.029–283.919)
Practising recycling of medication other than inhalers via disposal programme^a^						
No	149 (29.7)	8 (1.6)	157 (31.3)		NA	NA
Yes	119 (23.7)	226 (45.0)	345 (68.7)		NA	NA

AOR: adjusted odds ratio; CI: confidence interval; NA: not applicable; ND: not determined; OR: odds ratio; SD: standard deviation.

^a^ No. (%) reported if not otherwise stated.

^b^ SIGRE, the Spanish integrated management and collection system for pharmaceutical packaging programme.

^c^ The multivariate logistic regression model included those variables with an association in the bivariate analyses defined as P-value: < 0.1.

**Fig. 2:**
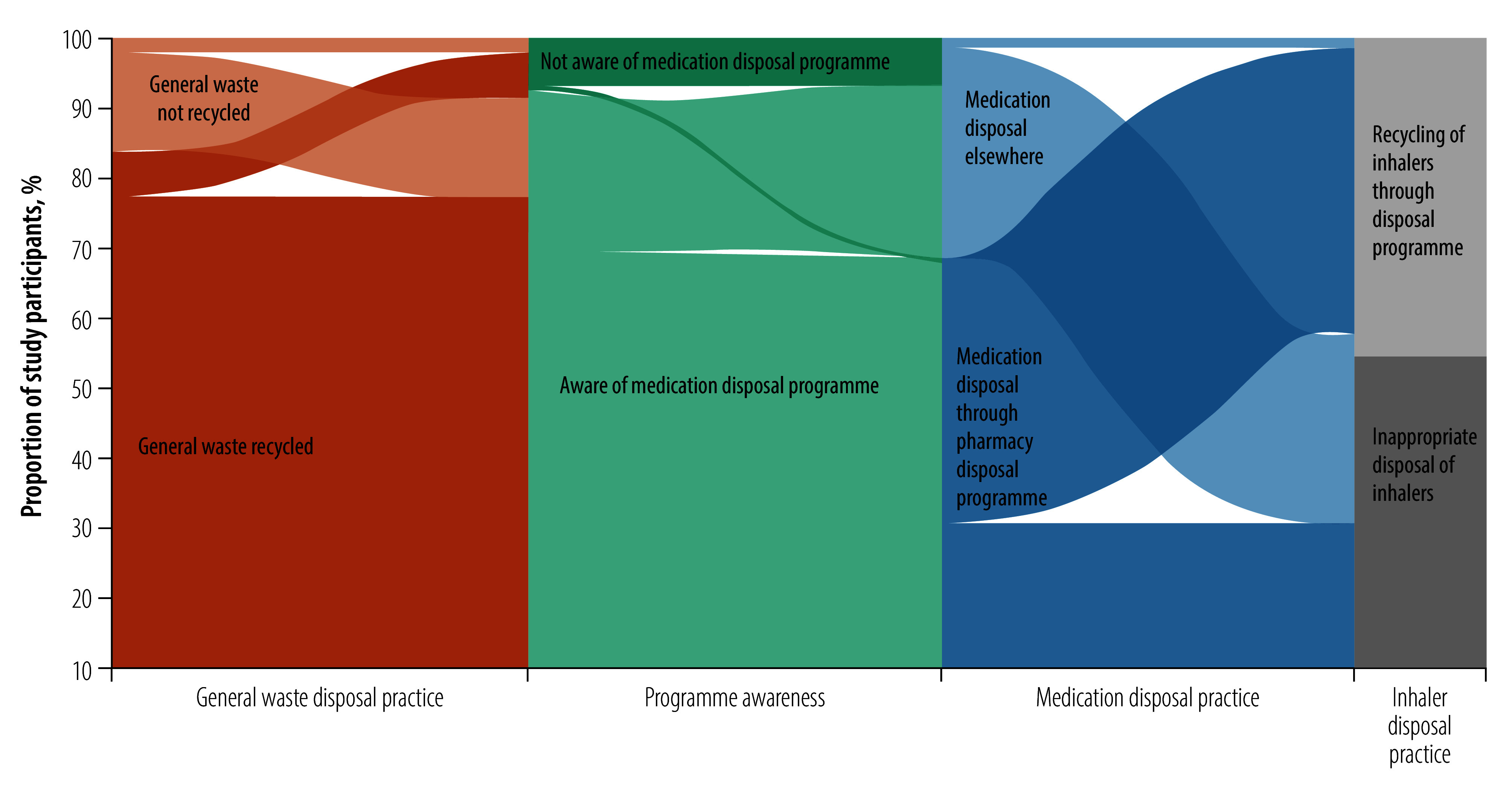
Correlation between environmental attitudes and the inhaler disposal practices of 502 study participants, Spain, March–November 2024

In terms of environmental awareness and practices, most patients (435; 86.7%) regarded environmental conservation as highly important and 351 (69.9%) believed that pollution and climate change had a negative effect on their health. With regards to general waste separation for recycling, 416 (82.9%) patients reported performing this regularly. When asked about the environmental impact of inhalers, 277 (55.2%) rated this as low; moreover, 336 (66.9%) patients believed inhalers had a lower environmental impact than other medications. Although 460 (91.6%) were aware of the medication disposal programme, only 234 (46.6%) correctly disposed of their inhalers. In contrast, 345 (68.7%) patients disposed of other medications appropriately. Our alluvial diagram (Fig. 2), in which the vertical dimension of each ribbon represents the proportion of study participants in that group, helps to visualize how the different combinations of general waste recycling practices and environmental awareness lead to the different types of inhaler disposal behaviour.

### Logistic regression modelling

We rejected both the presence of interactions and multicollinearity, with the exception of the variables “awareness of medication disposal programme” and “practising recycling of medication other than inhalers via disposal programme,” which were considered to be moderately collinear; the latter variable was therefore not included in the multivariate regression model. The only missing data was a single value for the variable “perception of environmental impact of inhalers relative to other medications.” We therefore did not perform any imputation process, as the proportion of missing data was negligible and unlikely to bias the results.

Results are reported in Table 1 as crude odds ratio (OR) and adjusted odds ratios (aOR) with 95% CI for the association between personal factors and inhaler waste disposal practices. In the crude analysis, sex, age, employment status, income, health problems limiting daily activities, practising general waste separation for recycling, perception of inhaler environmental impact and awareness of the medication disposal programme were associated with a higher likelihood of disposal at pharmacy collection points. In the adjusted model, low income (aOR: 2.628; 95% CI: 1.317–5.241), health problems limiting daily activities (aOR: 1.639; 95% CI: 1.057–2.540), practising general waste separation for recycling (aOR: 2.823; 95% CI: 1.617–4.926), perception of high inhaler environmental impact (aOR: 1.637; 95% CI: 1.104–2.428) and awareness of the medication disposal programme (aOR: 37.786; 95% CI: 5.029–283.919) remained associated with better inhaler disposal practices.

### Behaviour change

**Fig. 3:**
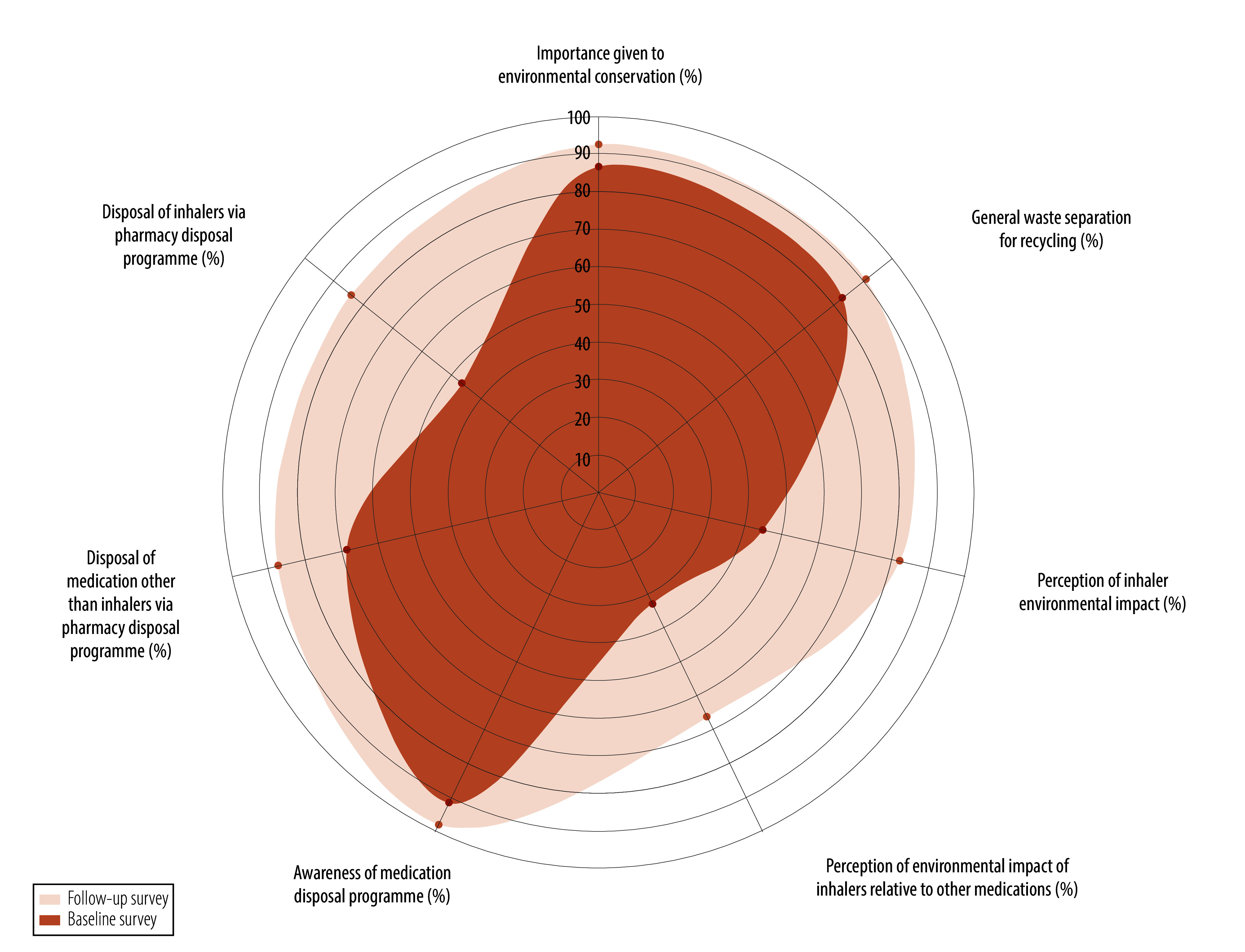
Environmental attitudes and medication disposal behaviour of inhalers of 502 participants before and after a pharmacy-led intervention, Spain, March–November 2024

Note: we conducted the follow-up survey 3 months after the intervention.

Following the pharmacist-led educational intervention, we observed significant improvements across all environmental awareness and behavioural variables after 3 months (*P*-value: < 0.001; Fig. 3). Correct inhaler disposal at pharmacy collection points increased from 46.6% (234/502) at baseline to 84.1% (418/497) after 3 months. Disposal of other medications at pharmacy collection points and household waste separation also increased, from 68.7% (345/502) to 87.5% (435/497) and from 82.9% (416/502) to 90.9% (452/497), respectively. Knowledge-related indicators showed marked gains: the number of patients who recognized the environmental impact of inhalers increased from 44.8% (225/502) to 82.1% (408/497); and the number of patients who correctly identified them as having a higher environmental impact than other medicines increased from 32.9% (165/502) to 66.6% (331/497). Awareness of the medication disposal programme rose from 91.6% (460/502) to 98.4% (489/497), and the number of respondents who attributed high importance to environmental concerns rose from 86.7% (435/502) to 92.8% (461/497).

## Discussion

The environmental impact of an inhaler is not defined solely by its propellant, but by its full life cycle from manufacture to disposal. Sustainable respiratory care therefore demands an integrated, evidence-based strategy, where clinical, environmental and structural dimensions align without compromising health outcomes.

Our study demonstrated that asthma patients expressed high concern for environmental conservation and recognized the potential health impact of climate change, as suggested elsewhere.^16^ However, despite almost all participants being aware of the medication disposal programme, less than half reported using it for inhaler disposal, as previously suggested.^14^ Our study also found that only one third of participants believed that inhalers had a higher environmental impact compared with other medicines, in line with previous results from Canada.^17^ In a previous study in Spain, only one third of asthma or chronic obstructive pulmonary disease (COPD) patients had been informed of appropriate methods of inhaler disposal.^14^ Our findings are therefore consistent with other international studies, indicating a widespread lack of knowledge among patients regarding the environmental impact of inhalers and available disposal pathways.^17^^–^^19^ The fact that two thirds of patients correctly discarded other medicines through the disposal programme but not inhalers, despite them being environmentally hazardous devices, reveals an educational blind spot in asthma patients.

In our adjusted analysis, prior awareness of the disposal programme emerged as the strongest correlate of appropriate inhaler disposal, underscoring the central role of targeted information in promoting environmentally responsible behaviour. Similarly, patients aware of the environmental impact of inhalers were significantly more likely to manage inhaler waste properly, aligning with previous evidence on the importance of environmental literacy in promoting sustainable health practices.^14^^,^^17^^,^^18^^,^^20^^–^^22^ Practising general waste separation in daily life also showed a robust association, suggesting that established pro-environmental habits may extend to medicine-related decisions.^23^^,^^24^ Lower income and limited daily activity were also linked to better practices, possibly the result of increased health-care interactions although further research is needed to explore these factors.

Our findings show that a brief counselling session, paired with a visual infographic, doubled the number of appropriate inhaler disposals. This result not only highlights the link between patient education and behaviour, but also demonstrates that pharmacist-delivered interventions can effectively change environmentally harmful habits.^22^^,^^25^ We achieved this through a simple, scalable action integrated into routine clinical workflows nationwide, underscoring the feasibility and real-life applicability of our study. Notably, our intervention also enhanced broader environmental awareness, including the importance of environmental conservation, general waste segregation and the carbon footprint of inhalers. These findings support the embedment of sustainability into clinical care, highlighting the role of health workers in decarbonizing health care, as well as their potential to drive broader shifts in environmental literacy and action through routine patient interaction.^17^^,^^18^^,^^26^

We acknowledge the persistence of well documented barriers to the correct disposal of medicines. Patients may prefer giving unused medicines to family, friends or charities rather than using other disposal methods.^21^ A key structural barrier is the absence or limited scope of medicine return systems in many countries, with variability in performance across systems if available.^20^^–^^27^ The most effective schemes, such as those in France, Portugal, Spain and Sweden, are extended producer responsibility systems, with national coverage and pharmacy-based collection points. Voluntary or government-led systems have shown poorer outcomes.^20^ In some countries, unclear procedures or a lack of available systems result in pharmacies refusing to accept returns.^19^ However, when accessible return schemes are available and the environmental impact is understood, patients report high satisfaction and willingness to dispose correctly.^22^

Achieving meaningful progress in inhaler sustainability demands a broader transformation beyond behavioural change and availability of medication disposal systems. Progress requires coordinated action across the entire therapeutic pathway, beginning at prescription. In Spain, a recent sustainable prescribing framework estimated that changing 50% of prescriptions for pressurized metered-dose inhalers to prescriptions for dry powder inhalers could prevent up to 200 000 tonnes of CO_2_-equivalent emissions annually.^28^ This Spanish prescription framework, along with other national initiatives, advocates a balanced approach where treatment decisions incorporate clinical, environmental and patient-centred considerations. Indiscriminate prescription switching may compromise adherence and asthma control,^29^ highlighting the importance of multidisciplinary collaboration to ensure that coordination and therapeutic continuity are preserved. Another reason for caution is that, although dry powder inhalers emit less greenhouse gas emissions, life-cycle assessments show they may be more harmful than pressurized metered-dose inhalers in terms of material depletion, water eutrophication, acidification, ecotoxicity and photochemical oxidants formation.^13^

At the industrial level, progress is needed in product design, such as promoting reusable components, integrating dose counters to prevent premature disposal. Over one half of patients struggle to determine whether their pressurized metered-dose inhalers are empty, often discarding them prematurely.^19^ The industry should also adopt standardized eco-labelling with information about environmental risks. Innovations should include new propellants, reusable plastic holders across multiple prescriptions, the minimization of packaging waste and increased availability of active ingredients across all inhaler device types.^30^^,^^31^

Our study had several strengths: (i) our nationally representative study population and real-life study design means that our intervention is adaptable to everyday clinical practice; (ii) our simple and brief intervention is highly conducive to inclusion in routine care settings; and (iii) by distributing our infographic in the four main languages of Spain (Spanish, Catalan, Galician and Basque), we allowed widespread access. However, a few limitations must also be considered. The relatively short follow-up period limited any long-term assessment of the effects of the intervention. Our study is also subject to potential self-reporting bias. A direct environmental outcome measure, for example actual number of inhalers returned, would offer more objective data, although the consistency of the observed associations and the concordant changes across related behavioural and knowledge variables after the intervention support the validity of the reported practices. Furthermore, although focused on patients with uncontrolled severe asthma, we do not anticipate major differences in results compared with those with milder asthma or COPD. Further evaluation of cultural and behavioural factors is warranted to improve interpretation and guide future strategies. Finally, although new lower global warming potential propellants, such as HFA-152a, HFO-1234ze, are under development, their implementation remains limited because of regulatory, technical and safety considerations, and their potential environmental and health trade-offs should be carefully assessed, reinforcing the need for complementary strategies such as appropriate prescribing and proper inhaler disposal.

Our multicentre study highlights the need to enhance inhaler waste management practices among asthma patients and demonstrates the effectiveness of pharmacist-led educational interventions in promoting environmental responsibility. Our findings reveal a notable discrepancy between the environmental concerns of patients and their actual inhaler disposal behaviour, largely because of limited knowledge of the environmental impact of inhalers; this knowledge gap can be substantially narrowed through brief, targeted interventions at the point of care.

## References

[1] State of the global climate 2024. Geneva: World Meteorological Organization; 2025. Available from: https://wmo.int/publication-series/state-of-global-climate-2024 [cited 2025 Aug 30].

[2] Allen MR, Dube OP, Solecki W, Aragón-Durand F, Cramer W, Humphreys S, et al. Framing and context. In: Masson-Delmotte V, Zhai P, Pörtner HO, Roberts D, Skea J, Shukla PR, et al., editors. Global warming of 1.5°C. An IPCC Special Report on the impacts of global warming of 1.5°C above pre-industrial levels and related global greenhouse gas emission pathways, in the context of strengthening the global response to the threat of climate change, sustainable development, and efforts to eradicate poverty. Cambridge and New York: Cambridge University Press; 2018. pp. 49–92.

[3] Operational framework for building climate resilient and low carbon health systems. Geneva: World Health Organization; 2023. Available from: https://iris.who.int/handle/10665/373837 [cited 2026 Apr 6].

[4] Keil M, Frehse L, Hagemeister M, Knieß M, Lange O, Kronenberg T, et al. Carbon footprint of healthcare systems: a systematic review of evidence and methods. BMJ Open. 2024 Apr 30;14(4):e078464. 10.1136/bmjopen-2023-078464 38688670 PMC11086491

[5] Karliner J, Slotterback S, Boyd R, Ashby B, Steele K. Health care’s climate footprint: how the health sector contributes to the global climate crisis and opportunities for action. Health Care Without Harm Climate-Smart Health Care Series Green Paper. No. 1. Reston: Health Care Without Harm 2019. Available from: https://global.noharm.org/sites/default/files/documents-files/5961/HealthCaresClimateFootprint_092319.pdf [cited 2026 Apr 6].

[6] Delivering a ‘net zero’ national health service. London: NHS England; 2022. Available from: https://www.england.nhs.uk/greenernhs/wp-content/uploads/sites/51/2020/10/delivering-a-net-zero-national-health-service.pdf [cited 2026 Apr 6].

[7] Marrauld L, Egnell M, Verneuil B, Rambaud T. Soigner les patients tout en soignant la planète: le bilan carbone du système de santé français et ses leviers de reduction. [Caring for patients while caring for the planet: the carbon footprint of the French healthcare system and levers for its reduction.] Med Mal Metab. 2023;17(4):318–25. French. 10.1016/j.mmm.2023.05.003

[8] Jongsma ME, Bogaards MJ, Grassi JB, van der Valk R, Egberts ATCG, Ossebaard HC. [Medication waste in a hospital setting: concerns and considerations.] Ned Tijdschr Geneeskd. 2022 Nov 14;166:D7044. Dutch. 36633070

[9] Woodcock A, Beeh KM, Sagara H, Aumônier S, Addo-Yobo E, Khan J, et al. The environmental impact of inhaled therapy: making informed treatment choices. Eur Respir J. 2022 Jul 21;60(1):2102106. 10.1183/13993003.02106-2021 34916263 PMC9301054

[10] La AEMPS informa sobre los propelentes utilizados en inhaladores presurizados y cómo reducir su huella de carbono. Ref: MUH 09. Madrid: Agencia Española de medicamentos y productos sanitarios (AEMPS); 2022. Spanish. Available from: https://www.aemps.gob.es/informa/notasInformativas/medicamentosUsoHumano/2022/docs/Nota%20Informativa_MUH-09-2022-inhaladores.pdf [cited 2026 Apr 6].

[11] Achebak H, Devolder D, Ingole V, Ballester J. Reversal of the seasonality of temperature-attributable mortality from respiratory diseases in Spain. Nat Commun. 2020 May 20;11(1):2457. 10.1038/s41467-020-16273-x 32433517 PMC7239891

[12] Chan EYY, Goggins WB, Yue JSK, Lee P. Hospital admissions as a function of temperature, other weather phenomena and pollution levels in an urban setting in China. Bull World Health Organ. 2013 Aug 1;91(8):576–84. 10.2471/BLT.12.113035 23940405 PMC3738307

[13] Jeswani HK, Azapagic A. Life cycle environmental impacts of inhalers. J Clean Prod. 2019;237:117733. 10.1016/j.jclepro.2019.117733

[14] de Simón Gutiérrez R, Ginel Mendoza L, Hidalgo Requena A, Rico Munilla D, Cantalapiedra Fernández F. [Do patients deliver inhalers correctly? The AIRA project.] Semergen. 2022 Jan-Feb;48(1):14–22. Spanish. 10.1016/j.semerg.2021.07.011 34479795

[15] Garin N, Zarate-Tamames B, Vaquero-Alvarez E, Gomez-Rivas P, Jornet-Montaña S, Del Estal-Jimenez J, et al. Supplementary material - Promoting sustainable respiratory care: inhaler waste management, behavioural factors and the role of health-care professionals [online repository]. London: figshare; 2026. 10.6084/m9.figshare.31959636

[16] Wilkinson A, Woodcock A. The environmental impact of inhalers for asthma: a green challenge and a golden opportunity. Br J Clin Pharmacol. 2022 Jul;88(7):3016–22. 10.1111/bcp.15135 34719810

[17] Quantz D, Wong GYC, Liang K. Patient perspectives on the environmental impact of inhalers: a survey in British Columbia. Can Pharm J. 2023 Oct 11;156(6):298–302. 10.1177/17151635231202980 38024456 PMC10655797

[18] Chakma MS, Usmani OS. Inhalers and the environment: pollution, plastics and policy. Pneumon. 2022;35(4):26. 10.18332/pne/154608

[19] Murphy AC, Carroll W, Gotsell M, Potter C, Quint JK, Malone R. How do patients determine when their inhaler is empty? Insights from an analysis of returned inhalers and a patient survey. BMJ Open Respir Res. 2024 Dec 25;11(1):e002579. 10.1136/bmjresp-2024-002579 39721746 PMC11751839

[20] Management of pharmaceutical household waste: limiting environmental impacts of unused or expired medicine. Paris: Organisation for Economic Co-operation and Development Publishing; 2022. 10.1787/3854026c-en

[21] AlAzmi A, AlHamdan H, Abualezz R, Bahadig F, Abonofal N, Osman M. Patients’ knowledge and attitude toward the disposal of medications. J Pharm (Cairo). 2017;2017:8516741. 10.1155/2017/8516741 29130019 PMC5654249

[22] Murphy A, Howlett D, Gowson A, Lewis H. Understanding the feasibility and environmental effectiveness of a pilot postal inhaler recovery and recycling scheme. NPJ Prim Care Respir Med. 2023 Jan 21;33(1):5. 10.1038/s41533-023-00327-w 36681666 PMC9864496

[23] Zsóka Á, Szerényi ZM, Széchy A, Kocsis T. Greening due to environmental education? Environmental knowledge, attitudes, consumer behavior and everyday pro-environmental activities of Hungarian high school and university students. J Clean Prod. 2013;48:126–38. 10.1016/j.jclepro.2012.11.030

[24] Watkins S, Barnett J, Standage M, Kasprzyk-Hordern B, Barden R. Household disposal of pharmaceuticals: attitudes and risk perception in a UK sample. J Mater Cycles Waste Manag. 2022;24(6):2455–69. 10.1007/s10163-022-01494-7

[25] Davis R, Campbell R, Hildon Z, Hobbs L, Michie S. Theories of behaviour and behaviour change across the social and behavioural sciences: a scoping review. Health Psychol Rev. 2015;9(3):323–44. 10.1080/17437199.2014.941722 25104107 PMC4566873

[26] MacNeill AJ, McGain F, Sherman JD. Planetary health care: a framework for sustainable health systems. Lancet Planet Health. 2021 Feb;5(2):e66–8. 10.1016/S2542-5196(21)00005-X 33581064

[27] Wang LS, Aziz Z, Wang ES, Chik Z. Unused medicine take-back programmes: a systematic review. J Pharm Policy Pract. 2024 Sep 9;17(1):2395535. 10.1080/20523211.2024.2395535 39257836 PMC11385643

[28] Garin N, Zarate-Tamames B, Lertxundi U, Martin da Silva I, Orive G, Crespo-Lessmann A, et al. The environmental impact of inhalers: a framework for sustainable prescription practices in Spain. Eur J Hosp Pharm. 2025;32:572–9. 10.1136/ejhpharm-2024-004402 39788701

[29] Nakanishi Y, Iwamoto H, Miyamoto S, Nakao S, Higaki N, Yamaguchi K, et al. Association between patient preference for inhaler medications and asthma outcomes. J Asthma Allergy. 2022 Oct 25;15:1539–47. 10.2147/JAA.S381509 36316999 PMC9617517

[30] Fulford B, Mezzi K, Whiting A, Aumônier S. Life-cycle assessment of the breezhaler® breath-actuated dry powder inhaler. Sustainability (Basel). 2021;13(12):6657. 10.3390/su13126657

[31] Keeley D, Scullion JE, Usmani OS. Minimising the environmental impact of inhaled therapies: problems with policy on low carbon inhalers. Eur Respir J. 2020 May 27;55(2):2001122. 10.1183/13993003.00048-2020 32108081

